# Duplication of the External Auditory Canal: Two Cases and a Review of the Literature

**DOI:** 10.1155/2012/924571

**Published:** 2012-11-19

**Authors:** John K. Goudakos, Sarantis Blioskas, George Psillas, Victor Vital, Konstantinos Markou

**Affiliations:** 1st Department of Otorhinolaryngology-Head and Neck Surgery, AHEPA University Hospital, Aristotle University of Thessaloniki, 54636 Thessaloniki, Greece

## Abstract

The objective of the present paper is to describe the clinical presentation, diagnostic process, surgical treatment, and outcome of 2 patients with first branchial cleft anomaly. The first case was an 8-year-old girl presented with an elastic lesion located in the left infra-auricular area, in close relation with the lobule, duplicating the external auditory canal. The magnetic resonance imaging revealed a lesion, appearing as a rather well-circumscribed mass within the left parotid gland and duplicating the ear canal. A superficial parotidectomy was subsequently performed, with total excision of the cyst. The second patient was a 15-year-old girl presented with a congenital fistula of the right lateral neck. At superficial parotidectomy, a total excision of the fistula was performed. During the operation the tract was recorded to lay between the branches of the facial nerve, extending with a blind ending canal parallel to the external acoustic meatus. Conclusively, first branchial cleft anomalies are rare malformations with cervical, parotid, or auricular clinical manifestations. Diagnosis of first branchial cleft lesions is achieved mainly through careful physical examination. Complete surgical excision with wide exposure of the lesion is essential in order to achieve permanent cure and avoid recurrence.

## 1. Introduction

 First branchial cleft anomalies are relatively uncommon congenital malformations of the head and neck, accounting for less than 10% of all branchial anomalies, and can be roughly classified as cysts, sinuses, and fistulae [1].

Clinically they may appear as chronic purulent ear drainage, periauricular swelling in the parotid area, or persistent fistula of the neck and may be present in both adults and children. First branchial cleft cysts of the parotid area can be a significant problem of differential diagnosis. Misdiagnosis has often led to inadequate management of the lesions and to a high percentage of recurrence. Clinical presentation, imaging characteristics, and knowledge of the anatomy and embryology of the area are important in limiting differential diagnosis.

The purpose of our paper is to report two cases of first branchial cleft anomalies, diagnosed and treated successfully in our clinic. Additionally, a literature review of the characteristics, the diagnostic evaluation, and treatment options of first branchial cleft anomalies is presented.

## 2. Case 1

 An 8-year-old girl was admitted to our department with a mass in her left parotid area, which was present for one month and has increased in size over the last 7-day period. On examination a tender mass was palpated in the left parotid area. The lesion was elastic, moderately movable, and mildly painful, located in the left infra-auricular area, in close relation with the lobule and external auditory canal.

**Figure 1:**
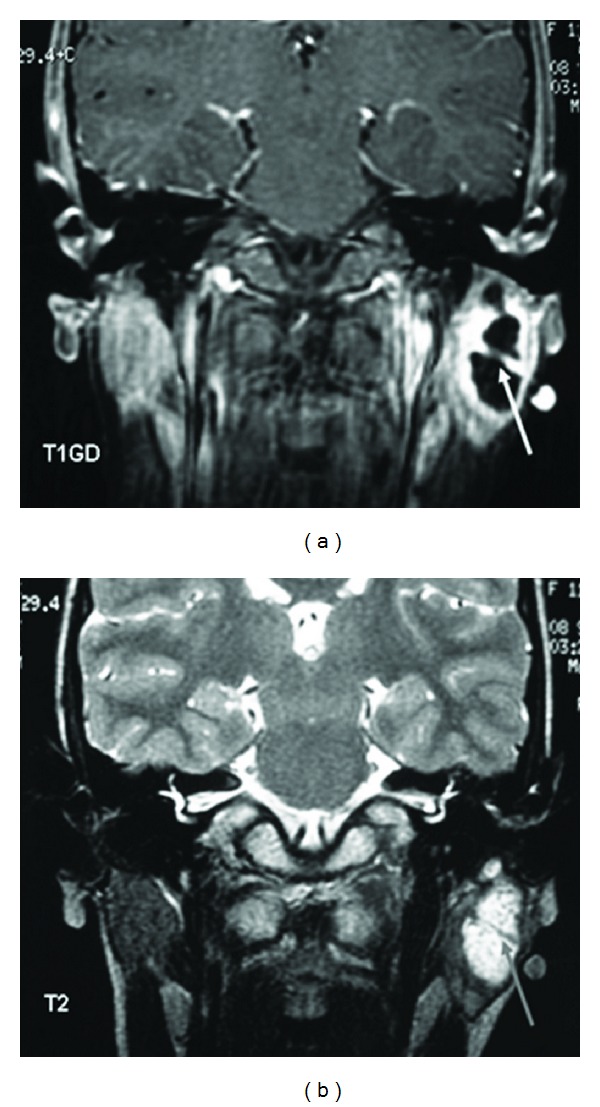
Coronal MR T1-contrast enhanced and T2-weighted images demonstrating the lesion in the left parotid gland (arrows: cyst with internal septum).

 The patient underwent magnetic resonance imaging (MRI) of the head and neck, demonstrating the anomaly (Figures 1(a) and 1(b)). The lesion appeared as a rather well-circumscribed mass in the left parotid area, within the parotid gland. Cystic origin was well established (high signal in T2-weighted sequence) and the presence of an internal septum was noted. In contrast, in enhanced T1 sequence (after intravenous gadolinium administration) both the wall of the lesion and the internal septum were enhanced. Moreover, the top side of the mass projects on the external auditory canal, duplicating the ear canal.

**Figure 2:**
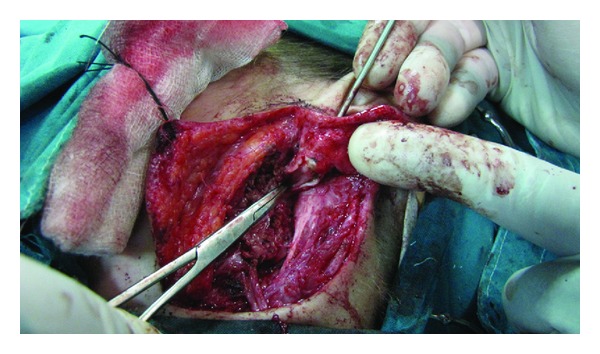
Exposure of the cyst, duplicating the cartilaginous part of the external auditory canal.

**Figure 3:**
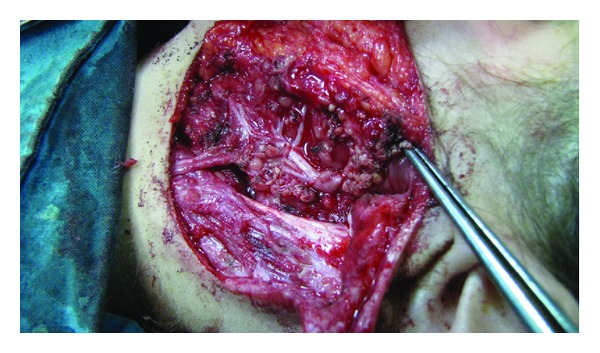
Superficial parotidectomy with complete excision of the cyst. Branches of the facial nerve exposed after performing a superficial parotidectomy.

A superficial parotidectomy was subsequently performed, with total excision of the cyst. During the operation it was confirmed that the cystic mass extended upwards, being in close contact with the cartilaginous part of the external auditory canal, but no communication with the canal or the middle ear was identified (Figure 2). The lesion lied superficially to the facial nerve, as we expected considering its embryological origin (Figure 3). The location of the facial nerve was identified anatomically with standard techniques and exposed with blunt dissection; electrophysiological monitoring throughout the procedure reduced the risk of injury. Once the facial nerve and its branches had been fully identified and carefully dissected, the anomaly was completely resected. 

Pathological examination of the surgical specimen showed skin, squamous epithelium, and cartilage, confirming the diagnosis of first branchial cleft cyst (type II).

No sign of recurrence was noted after a follow-up period of 15 months. 

## 3. Case 2

**Figure 4:**
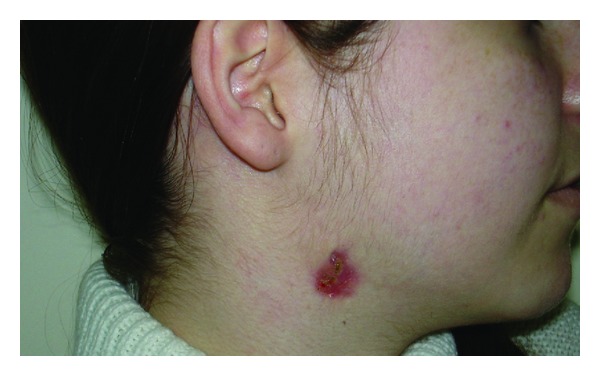
Fistula of the right lateral neck.

**Figure 5:**
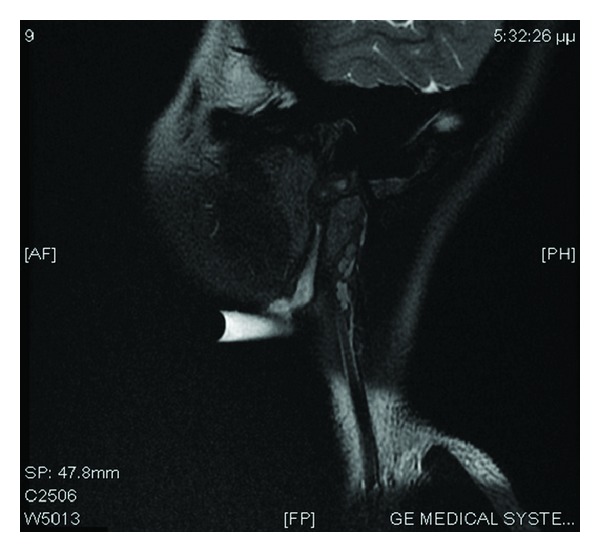
Lateral view T1-contrast enhanced MRI demonstrating the fistula and its extension to the acoustic meatus.

The second patient was a 15 year-old girl presented in our Department with a congenital fistula of the lateral neck with recurrent episodes of drainage during the last years (Figure 4). Patient's MRI (T1 weighted sequence—lateral view) revealed a fistula extending form right lateral neck up to the external acoustic meatus (Figure 5).

**Figure 6:**
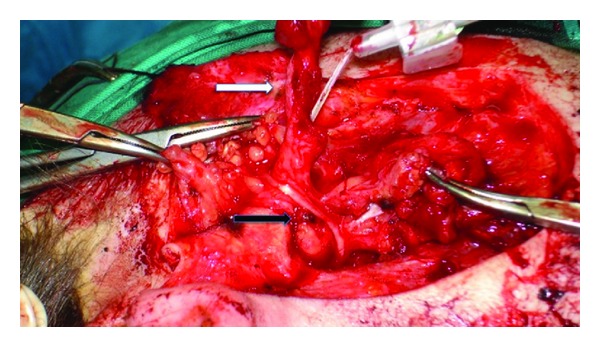
Superficial parotidectomy with complete excision of the fistula (white arrow—abbocath catheter), being between branches of the facial nerve exposed after performing a superficial parotidectomy (black arrow).

At superficial parotidectomy, a total excision of the fistula was performed. During the operation the tract was recorded to lay between the branches of the facial nerve, extending with a blind ending canal parallel to the external acoustic meatus (Figure 6). As in our first case, the facial nerve was identified anatomically with standard techniques and an electrophysiological monitoring throughout the procedure reduced the risk of injury. Once the facial nerve and its branches had been fully identified, the fistula was completely resected revealing the duplication of external acoustic meatus.

Pathological examination of the surgical specimen revealed that fistula has lined by squamous epithelium and contained cartilage and skin adnexa, indicating first branchial cleft anomaly. Granulomatous and giant cell reaction was also focally found.

No sign of recurrence was noted after a follow-up period of 12 months.

## 4. Discussion

 First branchial cleft anomalies are rare malformations which account for less than 10% of all branchial clefts anomalies and appear in both adults and children [1–3]. As parotid gland and facial nerve have a somewhat later embryological development, a first branchial anomaly has a variable relation to the parotid gland and facial nerve [4–6].

 First branchial cleft anomalies are the result of abnormal embryogenesis and arise from incomplete closure of the ventral portion of the first branchial cleft. Whether the defect is a fistula, sinus, or cyst depends on the degree of closure [5]. The anomaly begins on the floor of the external auditory canal in the cartilaginous portion or the bony-cartilaginous junction and ends somewhere in the submandibular region depending on the extend of the anomaly of the fusion [7].

The classification by Work is perhaps the most widely quoted. He classified these anomalies into two types based on clinical and histological features [8]. Type I anomalies present as a cystic mass of ectodermal origin, lined by squamous epithelium with or without accessory skin structures. Type I lesions occur medial to the concha, often extending into the postauricular crease and end in a cul-de-sac at the bony plate at the level of the mesotympanum. Type II anomalies present as cysts, sinuses, or fistulae, of both ectodermal and mesodermal origin, containing squamous epithelium with adnexal skin structures or cartilage. They may pass through the parotid gland in a variable relation to the facial nerve, indicating a disturbance of fusion in the arch itself and the sinus tract may end either in the cartilaginous external auditory canal or extend to the face or upper neck. According to this classification, either type is regarded as duplication of either the membranous (type I) or both the membranous and cartilaginous (type II) portions of the external auditory canal. 

 Depending on the type of the anomaly, clinical manifestations can be roughly divided in cervical, parotid, and auricular symptoms [7]. Cervical symptoms consist of a pit-type depression near the angle of the mandible, which produces a string of clear material when pressured. The symptomatology of our first case included of parotid signs as a small tumor-like mass located in the parotid gland was present, while cervical manifestations presented in our second patient. Auricular symptoms consist of otorrhea with mucopurulent or purulent discharge, depression, pit, or mass in the external auditory canal and often periauricular swelling. There may have been a history of recurrent otitis externa, or hearing loss. 

Diagnosis of first branchial cleft lesions is achieved mainly through careful physical examination of both the neck and the external auditory canal. According to Triglia et al. physical examination of the external auditory canal allowed discovery of a fistula in 44% of the patients, while an asymptomatic membranous attachment between the floor of the external auditory canal and the tympanic membrane was found in 10% of the cases [7]. Such myringeal webs are significant diagnostically, not only because they are easily discovered but also because their presence can be pathognomonic of type II first branchial cleft lesions [9]. Ultimately, thorough knowledge of the anatomy and embryology of the area combined with a high degree of clinical suspicion appear to be the key factors of an early and accurate diagnosis.

 Radiological examinations can be helpful diagnostically, in order to determine the typical anatomical localisation, the extent, the size, and the relationship of the lesions to surrounding structures. MRI allows assessment of the extent of the anomaly, especially in the parotid area, and high-resolution-computed tomography imaging shows its exact relationship with the external auditory canal and the middle ear. Fistulas and sinuses are not always obvious on CT and a fistulogram can be useful [10]. 

 We cannot stress enough the importance of clinical suspicion on initial presentation of such lesions. Misdiagnosis and subsequent inappropriate or inadequate treatment are unfortunately still the rule. The delay between initial presentation and adequate treatment is reported to reach 3.5 to 4 years [3, 7].

 Differential diagnosis of first branchial cleft anomalies must be made mainly with second branchial cleft anomalies. Parotid branchial cleft cysts, in particular, must be differentiated from any cystic mass of the parotid [11, 12]. Retention cysts, posttraumatic sialoceles, lymphangiomas, benign, and malignant parotid tumors must all be considered.

Complete surgical excision with wide exposure of the lesion is essential in order to achieve permanent cure and avoid recurrence. Parotidectomy and exposure of the facial nerve are required in the vast majority of cases [7]. Any sinus, fistula, or involved area with skin breakdown should be excised in continuity. In some cases, resection of a small portion of the skin and cartilage of the external auditory meatus is necessary to prevent recurrence.

 The overall recurrence rate after operation for branchial cysts is 3 to 5%, with a slight increase for sinuses and fistulae [13, 14]. Recurrence rate is further increased to 20% in case of previous infection before the operation [15].
